# Clinical application of capsule endoscopy in children with small bowel disease in Wuhan, China

**DOI:** 10.3389/fphys.2025.1714118

**Published:** 2026-01-13

**Authors:** Pingping Xu, Dan Luo, Baoxiang Wang, Yingru Liu, Siyue Tang, Shan Guo, Zhi Yu, Leyong Zheng, Jie Feng, Huifen Huang, Yuan Gao, Fang Wang, Wei Su

**Affiliations:** 1 Department of Gastroenterology/Center of Digestive Endoscopy of Wuhan Children’s Hospital, Tongji Medical College, Huazhong University of Science and Technolog, Wuhan, China; 2 Medical Department of School of Medicine, Wuhan University of Science and Technology, Wuhan, China; 3 Joint Training Base at The Postgraduate Level From Wuhan University of Science and Technology and Wuhan Children’s Hospital, Wuhan, China; 4 Health Management College of Xianning Vocational and Technical College, Xianning, China

**Keywords:** capsule endoscopy, children, clinical application, pediatric CE, small intestinal diseases

## Abstract

**Objective:**

To evaluate the clinical application value of capsule endoscopy (CE) in the diagnosis of small intestinal diseases in children in Wuhan.

**Methods:**

A retrospective analysis was conducted on the clinical data of children who underwent CE examination at the Digestive Endoscopy Center of Wuhan Children’s Hospital from July 2021 to November 2024. The completion rate of CE examination, disease detection rate, small intestinal transit time, and adverse reactions were analyzed.

**Results:**

Among the 151 children, 97 were male (64.24%) and 54 were female (35.76%), with an average age of 10.9 years. CE was swallowed orally in 133 cases (88.08%) and placed through gastroscopy in 18 cases (11.92%). Complete small intestinal examination was achieved in 144 cases (95.36%), with an average small intestinal transit time of 4 h and 11 min for those swallowed orally and 4 h and 16 min for those placed through gastroscopy. Seven cases (4.64%) did not complete the full small intestinal examination. Abdominal pain (99 cases, 65.56%), anemia (19 cases, 12.58%), and hematochezia and melena (23 cases, 15.23%) were the most common indications for examination. Among the 151 children, 116 (76.82%) had positive results, including 68 cases of nonspecific small intestinal inflammation, 29 cases of Crohn’s disease, 4 cases of Henoch-Schönlein purpura, 6 cases of Meckel’s diverticulum, 4 cases of small intestinal parasitic disease, 2 cases of collagenous gastritis, 1 case of melanocytic polyp, 1 case of lymphangiectasia, and 1 case of blue rubber bleb nevus syndrome. Nonspecific small intestinal inflammation was the most common in all age groups, while Crohn’s disease was mainly seen in the 7–12 years and 13–18 years age groups, accounting for 55.17% and 44.83%, respectively. No adverse reactions occurred in all children.

**Conclusion:**

CE examination in children in Wuhan has a high disease detection rate and good safety. CE can be further promoted in the diagnosis of small intestinal diseases in children.

## 1 Introduction

As the longest organ of the digestive system, small intestine has three parts (i.e., duodenum, jejunum and ileum) and is an important site for food digestion and absorption. The small intestine lies deep in the abdominal cavity, with about 4–6 m in length, including multiple curves and overlaps, and has been historically seen as the “black box” for gastrointestinal examination (Iwama et al., 2023; Singeap et al., 2023). Traditional enteroscopy is invasive, requires anesthesia, and has the risk of postoperative abdominal pain, bleeding and perforation. Especially, children have narrow intestinal lumen and thin intestinal wall, which increase the difficulty in performing enteroscopy routinely among pediatrics (Zhao et al., 2022). By using a pill-sized capsule containing a camera, capsule endoscopy (CE) is a minimally invasive diagnostic procedure with high lesion detection rate, safety and simple operation, and has shown significant advantages in the diagnosis of small intestinal diseases in recent years (Iwama et al., 2023; Kim and Chun, 2021; Melson et al., 2021).

Global epidemiological data show that the most common indications for pediatric CE vary by region but are consistently dominated by chronic abdominal pain (accounting for 40%–70% of total indications), followed by unexplained gastrointestinal bleeding (hematochezia/melena, 15%–30%), unexplained iron-deficiency anemia (10%–20%), and chronic diarrhea (5%–10%) (Ashton and Beattie, 2024; Légeret et al., 2022; Wu et al., 2020). Rare indications include suspected small intestinal structural abnormalities (e.g., Meckel’s diverticulum) or systemic diseases involving the small intestine (e.g., Henoch-Schönlein purpura) (Kaul et al., 2024; Oka et al., 2023). In Asian pediatric populations, chronic abdominal pain and unexplained anemia are the top two indications for CE, accounting for 55%–70% and 10%–15% respectively, which is slightly different from Western countries where gastrointestinal bleeding is the primary indication (Liao et al., 2024; Yang et al., 2022). While CE use in adults is well-established, its specific application in the pediatric population of Wuhan, China, remains unreported. By retrospectively analyzing 151 pediatric patients undergoing CE in Wuhan Children’s Hospital, this study investigated the clinical application and safety of CE in the diagnosis of small bowel disease, in order to lay a foundation for the further popularization and application of CE in the field of pediatric digestion.

## 2 Materials and methods

### 2.1 Ethical statement

This study received approval from the Ethics Committee of Wuhan Children’s Hospital (No. 2025R051-E01), and informed consent was obtained from the patient’s families of the patient for access to clinical data.

### 2.2 Study population

A total of 151 pediatric patients undergoing CE were retrospectively recruited from July 2021 to November 2024 in the Center of Digestive Endoscopy of our hospital. Inclusion criteria were as follows: (1) aged 2–18 years; (2) high suspicion of small bowel disease or other disease involving small bowel; (3) patients and/or their guardian signed consent form. Patients were excluded if (1) they had no surgical conditions or refused surgery; (2) they had confirmed or suspected gastrointestinal obstruction, stenosis or fistula; (3) they received cardiac pacemaker or other electronic instruments implantation; (4) they had structural swallowing disorders; (5) they refused CE due to various reasons.

### 2.3 Procedure

#### 2.3.1 Instrument

PillCam SB3 capsule endoscopy system (Given Imaging Ltd.) was used, including capsule, real-time recording device and workstation with RAPID Software. CE has diameter of 11 mm, length of 26 mm, weight of 2.89 g, one camera, endurance of 13–15 h, and operating temperature of 20 °C–45 °C.

#### 2.3.2 Examination process

##### 2.3.2.1 Gastrointestinal tract preparation

(1) Diet: patient was given liquid diet 1 day before the examination, and strict fasting after 8:00 p.m. before the examination was required; (2) Bowel preparation: at 6:00 p.m. before the examination and at 6:00 a.m. on the day of the examination, 25 mL/kg polyethylene glycol electrolyte powder solution (maximum 2 L) was orally administered, which was completed in 1h; (3) defoaming agent: 10 mL simethicone was orally administered after oral bowel cleansing agent on the day of the examination; (4). During the administration of polyethylene glycol electrolyte powder solution and simethicone, the child was advised to walk back and forth moderately and receive gentle abdominal massage to promote bowel peristalsis and improve bowel preparation efficacy.

##### 2.3.2.2 Adaptive swallowing training

(1) We first collected information on the child’s usual experience with swallowing oral medications (e.g., tablets, capsules, or syrups) to preliminarily assess their swallowing ability and predict potential difficulties in swallowing the CE capsule.

(2) For children with poor swallowing or aged 2–10 years, adaptive swallowing training was performed 1 day before the examination, that is, tried to swallow colloidal soft sugar with a volume from small to large (maximum CE size), so that the capsule could be swallowed smoothly.

##### 2.3.2.3 Swallowing CE

On the day of the examination, the child entered the CE examination room accompanied by a guardian, lay supine on the examination bed, and loosened or removed clothing around the abdomen to facilitate the placement of the sensor belt. Connected leads to the abdomen and placed a sensor belt to record images, and then the patient swallowed CE with a small cup of warm water in a sitting position.

##### 2.3.2.4 Monitoring

During the examination, real-time monitoring focused on the passage of CE through two key anatomical sites: the pylorus and the ileocecal orifice. (1) After the child swallowed the CE, the endoscopist confirmed its rapid entry into the gastric lumen via real-time imaging, then instructed the child to assume the right lateral decubitus position to promote gastric emptying. CE typically entered the duodenum from the gastric lumen 1–1.5 hrs later, after which the child was allowed to move freely to accelerate CE transit through the small intestine. (2) CE’s progress in the small intestine was monitored every 1 hr. The child was permitted to consume clear liquids 2 hours after CE ingestion and a light diet 4 hrs later. Between 4–6 hrs post-ingestion, real-time monitoring was performed to determine whether CE had entered the colon. (3) Completion of the examination was defined as CE traversing the entire small intestine and entering the colon; subsequent stool output was closely observed to confirm the natural excretion of the capsule. (4) After the examination, image data were downloaded from the recorder to the RAPID workstation, and two gastroenterologists independently analyzed the images to ensure diagnostic accuracy.

##### 2.3.2.5 Endoscopic placement of CE

Painless endoscopic placement of CE was conducted for children who failed to swallow capsules. The specific process was as follows: (1) The child was placed in the left lateral decubitus position, and routine gastroscopy was performed first under intravenous anesthesia (propofol); (2) (2) Fixed CE at the anterior end of the gastroscope with a snare, and CE was placed below the descending duodenum. The anesthesiologist participated throughout the procedure with concurrent vital signs monitoring.

### 2.4 Observation indicators

Operational definitions: (1) Completion of examination: CE traverses the entire small intestine (from duodenum to ileocecal valve) and enters the colon within the device’s battery life (13–15 h). (2) Non-completion of examination: CE fails to reach the ileocecal valve within 13–15 h due to factors such as intestinal stenosis, slow transit, or battery depletion.

Observation indicators included: (1) completion rate of small bowel examination by CE; (2) gastric transit time (GTT) and small bowel transit time (SBTT); (3) detection of small intestinal lesions and related diseases; (4) tolerance and complications.

## 3 Results

### 3.1 General information

**FIGURE 1:**
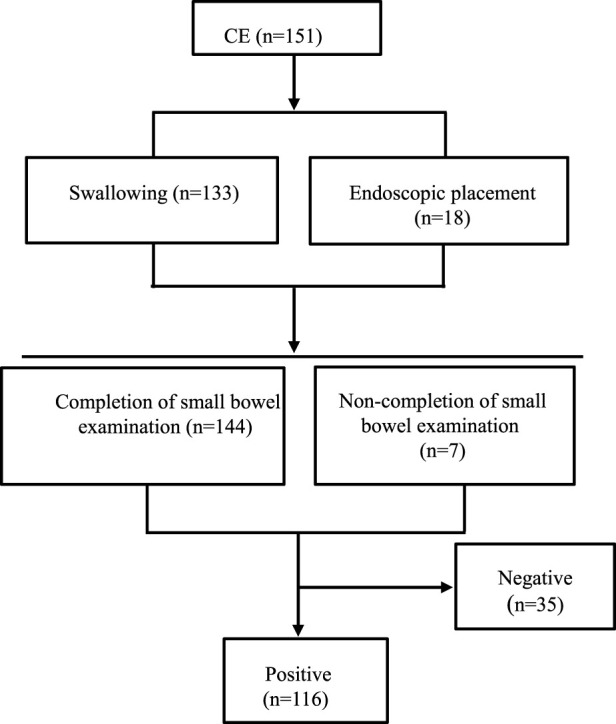
Flowchart of CE for 151 pediatric patients.

**FIGURE 2:**
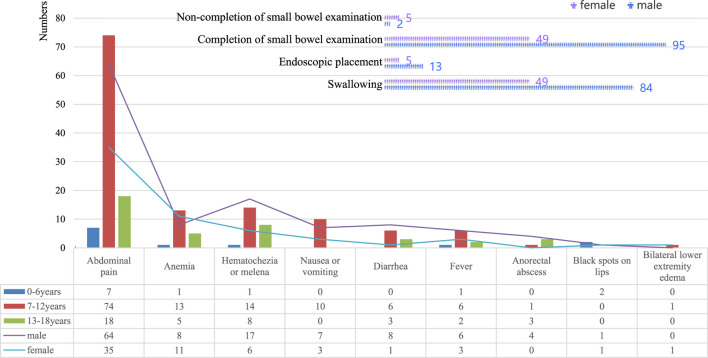
Demographics and clinical features of 151 pediatric patients.

A total of 151 pediatric patients were finally recruited, including 97 (64.24%) boys and 54 (35.76%) girls, with an average age of 10.9 years (ranging from 4.5–17.3 years). The lowest height and weight were 108 cm and 14 kg, separately. CE placement: 133 patients by swallowing (minimum age 4.5 years) and 18 patients by endoscopic placement (maximum age 14.5 years). As shown in Figure 1, 144 completed the whole small bowel examination. Reasons for CE examination were clearly defined as follows:①Chronic abdominal pain (duration ≥2 weeks, no obvious cause after routine examination): 99 cases (65.56%); ②Unexplained anemia (iron-deficiency anemia with hemoglobin <110 g/L in children <12 years or <120 g/L in children ≥12 years, no cause found by routine blood, stool, and abdominal ultrasound): 19 cases (12.58%); ③Hematochezia/melena (visible blood in stool or tarry stool, excluding anorectal diseases): 23 cases (15.23%); ④Chronic diarrhea (duration ≥2 weeks, ≥3 loose stools per day): 9 cases (5.96%); ⑤Unexplained fever (fever ≥38.5 °C for ≥1 week, no infection source identified): 9 cases (5.96%); ⑥Anorectal abscess (suspected fistula involving small intestine): 4 cases (2.65%); ⑦Lip melanotic spots (suspected Peutz-Jeghers syndrome): 2 cases (1.32%); ⑧Bilateral lower extremity edema (suspected hypoproteinemia due to small intestinal malabsorption): 1 case (0.66%). See Table 1 and Figure 2 for details.

**TABLE 1:** Demographics and clinical features of 151 pediatric patients.

General information	Cases (M/F)	0–6 years (M/F)	7–12 years (M/F)	13–18 years (M/F)
N (M/F)	151 (97/54)	10 (5/5)	105 (66/39)	36 (26/10)
Mean age (years)	10.9	5.6	10.3	14.0
Swallowing	133 (84/49)	5 (2/3)	94 (58/36)	34 (24/10)
Endoscopic placement	18 (13/5)	5 (3/2)	11 (8/3)	2 (2/0)
Completion of small bowel	144 (95/49)	10 (5/5)	102 (65/37)	32 (25/7)
Non-completion of small bowel	7 (2/5)	0	3 (1/2)	4 (1/3)
Chief complaint				
Abdominal pain	99 (64/35)	7 (4/3)	74 (45/29)	18 (15/3)
Anemia	19 (8/11)	1 (0/1)	13 (5/8)	5 (3/2)
Hematochezia or melena	23 (17/6)	1 (0/1)	14 (11/3)	8 (6/2)
Nausea or vomiting	10 (7/3)	0	10 (7/3)	0
Diarrhea	9 (8/1)	0	6 (5/1)	3 (3/0)
Fever	9 (6/3)	1 (1/0)	6 (3/3)	2 (2/0)
Anorectal abscess	4 (4/0)	0	1 (1/0)	3 (3/0)
Black spots on lips	2 (1/1)	2 (1/1)	0	0
Bilateral lower extremity edema	1 (0/1)	0	1 (0/1)	0

### 3.2 Completion of small bowel examination by CE

Among the 133 patients who swallowed the capsule, 6 failed to complete the small bowel examination due to CE battery depletion. At the time of battery failure, the capsule was located in the jejunum (n = 3) or ileum (n = 3); prolonged transit time was attributed to mild intestinal mucosal edema and delayed peristalsis (no evidence of capsule impaction or intestinal stenosis was found). Among the 18 patients who underwent endoscopic placement, 1 did not complete the examination (capsule remained in the distal ileum at battery depletion, associated with localized inflammatory stenosis in a child with Crohn’s disease). In summary, completion rate of small bowel examination by CE was 95.36% (Figure 1).

### 3.3 SBTT of CE

Among the 144 patients with complete examination, SBTT and GTT of 127 children who swallowed the capsule was 4h11min and 1h5 min, separately. On the other hand, SBTT of 17 children who underwent endoscopic placement of capsule was 4h16 min.

### 3.4 Diseases detected by CE

#### 3.4.1 Diseases detected by CE

**FIGURE 3:**
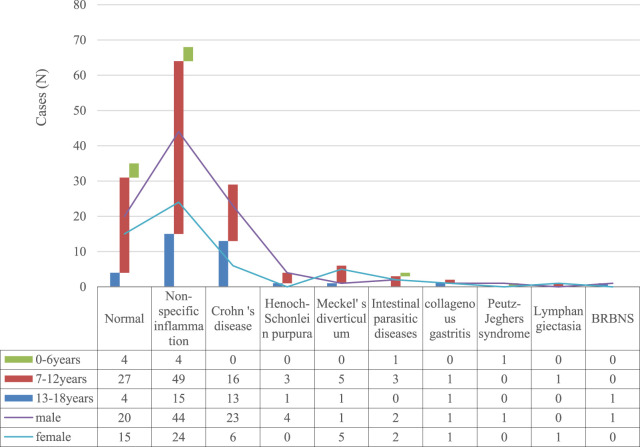
Diseases detected by CE among 151 pediatric patients.

**FIGURE 4:**
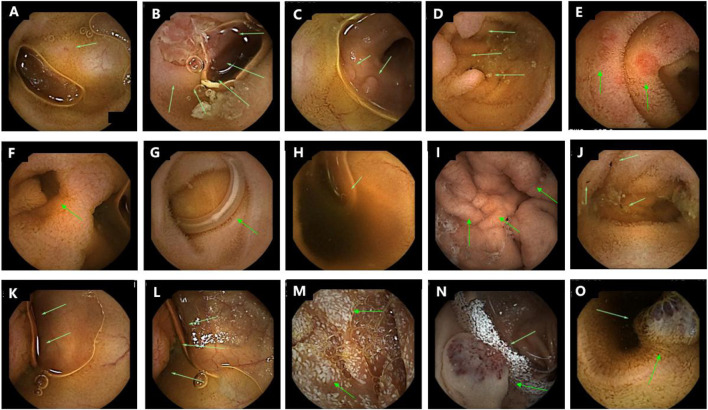
Typical imaging manifestations of detected diseases by CE. **(A)** Mild inflammation of small intestinal mucosa; **(B–D)** Large ulcers and multiple inflammatory hyperplasia bulges in small intestinal mucosa (Crohn‘s disease, typical skip lesions, deep mucosal ulcers, and focal inflammatory hyperplasia—confirmed by histopathological examination of subsequent enteroscopy biopsies; lymphatic hyperplasia was excluded based on clinical and pathological correlation); **(E)** Intestinal mucosal congestion and erosion (Henoch-Schonlein purpura); **(F)** Small bowel diverticulitis (Meckel’s diverticulum); **(G,H)** *Ascaris lumbricoides* and enterobius vermicularis (parasitic diseases); **(I,J)** Abnormal bulge of gastric mucosa fold, old hemorrhage in intestinal lumen, mild inflammation of local mucosa; **(K,L)** Intestinal polyp (Peutz-Jeghers syndrome); **(M)** Snowflake mucosa of small intestine (lymphangiectasia); **(N,O)** Blue-purple blebs in gastric and small intestinal mucosa (BRBNS).

A total of 116 positive results were reported, with a detection rate of 76.82%. As shown in Table 2 and Figures 3, 4, detected diseases included: 68 intestinal non-specific inflammation, 29 Crohn‘s disease, 4 Henoch-Schonlein purpura, 6 Meckel’s diverticulum, four intestinal parasitic diseases, two collagenous gastritis, 1 Peutz-Jeghers syndrome, one lymphangiectasia, and one blue rubber bleb nevus syndrome (BRBNS). Disease distribution varied by age group: in children aged 0–6 years, the most common findings were normal small bowel mucosa (n = 15) and non-specific inflammation (n = 7); in the 7–12 years group, normal mucosa (n = 28), non-specific inflammation (n = 42), and Crohn’s disease (n = 16) were the top three findings; in the 13–18 years group, non-specific inflammation (n = 19) and Crohn’s disease (n = 13) predominated, with normal mucosa accounting for only 8 cases. See Table 2 for details.

**TABLE 2:** Diseases detected by CE among 151 pediatric patients.

N (male/Female) CE results	Total	0–6 years	7–12 years	13–18 years
Normal	35 (20/15)	4 (1/3)	27 (17/10)	4 (2/2)
Non-specific inflammation	68 (44/24)	4 (2/2)	49 (31/18)	15 (11/4)
Crohn‘s disease	29 (23/6)	0	16 (13/3)	13 (10/3)
Henoch-schonlein purpura	4 (4/0)	0	3 (3/0)	1 (1/0)
Meckel’s diverticulum	6 (1/5)	0	5 (1/4)	1 (0/1)
Intestinal parasitic diseases	4 (2/2)	1 (1/0)	3 (1/2)	0
Collagenous gastritis	2 (1/1)	0	1 (0/1)	1 (1/0)
Peutz-jeghers syndrome	1 (1/0)	1 (1/0)	0	0
Lymphangiectasia	1 (0/1)	0	1 (0/1)	0
BRBNS	1 (1/0)	0	0	1 (1/0)
Total	151(97/54)	10(5/5)	105(66/39)	36(26/10)

Location and Nature of Lesions: (1) Nonspecific small intestinal inflammation (68 cases): 52 cases (76.47%) involved the jejunum and ileum, 12 cases (17.65%) the ileum alone, and 4 cases (5.88%) the jejunum alone; lesions were characterized by mucosal hyperemia, edema, and focal erosion; (2) Crohn’s disease (29 cases): 21 cases (72.41%) had ileal lesions, 6 cases (20.69%) ileal-colonic involvement, and 2 cases (6.90%) jejunal-ileal involvement; typical manifestations included skip lesions, deep ulcers, and inflammatory hyperplasia (confirmed by colonoscopy and pathology); (3) Henoch-Schönlein purpura (4 cases): All involved the jejunum and ileum, with mucosal congestion, erosion, and petechiae; (4) Meckel’s diverticulum (6 cases): All located in the terminal ileum, with diverticular wall inflammation and mucosal hyperemia; (5) Parasitic diseases (4 cases): Ascariasis (2 cases) and enterobiasis (2 cases), all in the jejunum, with visible worm bodies on imaging; (6) Other diseases: Collagenous gastritis (gastric body mucosal fold thickening), Peutz-Jeghers syndrome (ileal polyps), lymphangiectasia (jejunal “snowflake-like” mucosa), and BRBNS (gastric and jejunal blue-purple blebs) (Figure 4).

#### 3.4.2 Abdominal pain

**FIGURE 5:**
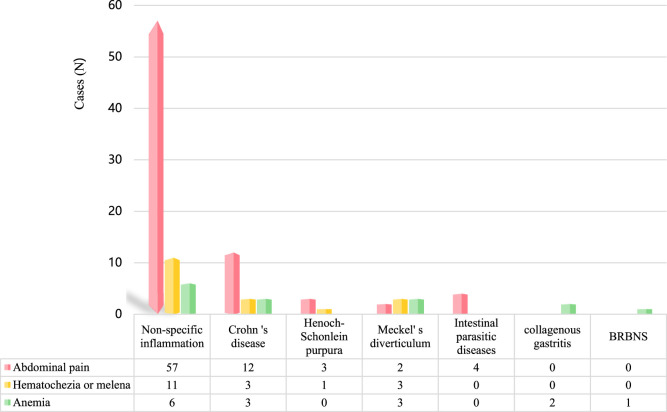
Disease detection of common Indications for CE.

Among 99 children with chief complaint of abdominal pain, small intestinal lesions were identified in 78 cases, with a detection rate of 78.79%. Related diseases included: 57 intestinal non-specific inflammation, 12 Crohn‘s disease, 3 Henoch-Schonlein purpura, 2 Meckel’s diverticulum, and four intestinal parasitic diseases. See Table 3 and Figure 5 for details.

**TABLE 3:** Disease detection of common indications for CE.

Symptoms detection results(n)	Abdominal pain	Hematochezia or melena	Anemia
CE cases	99	23	19
Positive results (positive rate)	78 (78.79%)	18 (78.26%)	15 (78.95%)
Non-specific inflammation	57	11	6
Crohn‘s disease	12	3	3
Henoch-schonlein purpura	3	1	0
Meckel’s diverticulum	2	3	3
Intestinal parasitic diseases	4	0	0
Collagenous gastritis	0	0	2
BRBNS	0	0	1

#### 3.4.3 Hematochezia or melena

Among 23 children with chief complaint of hematochezia or melena, small intestinal lesions were identified in 18 cases, with a detection rate of 78.26%. Related diseases included: 11 intestinal non-specific inflammation, 3 Crohn‘s disease, 1 Henoch-Schonlein purpura, and 3 Meckel’s diverticulum. See Table 3 and Figure 5 for details.

#### 3.4.4 Anemia

Among 19 children with chief complaint of anemia, small intestinal lesions were identified in 15 cases, with a detection rate of 78.95%. Related diseases included: six intestinal non-specific inflammation, 3 Crohn‘s disease, 3 Meckel’s diverticulum, two collagenous gastritis, and 1 BRBNS. See Table 3 and Figure 5 for details.

### 3.5 Tolerance and complications

CE was well tolerated in all children and was excreted from spontaneous defecation within 72 h after the examination without adverse reactions such as CE retention.

## 4 Discussion

Digestive endoscopy can directly observe the changes of gastrointestinal mucosa and is the gold standard for the diagnosis of gastrointestinal mucosal lesions (Feng and Liu, 2018). CE was first introduced in Israel in 2000 and achieved safe, accurate and noninvasive whole small intestinal endoscopic imaging for the first time. With advantages of flexibility and convenience, CE is an important diagnostic means of small intestinal diseases (Iddan et al., 2000; Cao et al., 2024; Wu et al., 2020; Ahmed, 2022) and is regarded as an significant milestone in the development of digestive endoscopy (Liao and Li, 2020; Xu, 2017). In 2004 and 2009, Food and Drug Administration (FDA) approved the application of CE among children aged ≥10 years and ≥2 years, respectively (Ahmed, 2022). Current reports of CE in children are limited, so this paper aims to investigate the clinical application of CE in children in Wuhan, China for the first time, as well as to provide an objective basis for the promotion of CE in this region.

School-aged children and adolescents mainly constituted our study population, with an average age of 10.9 years, which was is equivalent to the previous study (8.3–14 years) (Liao et al., 2024; Yang et al., 2022). Besides, there was more boys than girls, with a ratio of 1.8:1, which was similar with statistics in Zhejiang Province (1.87:1) and Shanghai (1.97:1) (Wu et al., 2020; Ma et al., 2009), but was lower than Xi’an (2.23:1) (Yang et al., 2022). Consistent with reports from Shanghai, abdominal pain was the most frequent indication of CE in our study, followed by anemia, hematochezia or melena, vomiting, diarrhea, fever, and anorectal abscess, but unlike adults whose main indication was gastrointestinal bleeding (Wu et al., 2020; Mejía et al., 2024; Cortegoso Valdivia et al., 2022). CE is the first-line diagnostic tool for evaluating small intestinal bleeding (Pennazio et al., 2023), and the difference in indications between children and adults may lie in the different disease spectra.

Completion of CE refers to the capsule traversing the entire small bowel and reaches the cecum (Wu et al., 2016). The potential reasons for the higher completion rate in our study (95.36%) compared with Guangzhou (89.8%) and Zhejiang (90%) may be related to differences in disease severity and etiological composition. For example, among the 7 non-completing cases in our study, only 1 had Crohn’s disease (mild intestinal inflammation without stenosis), while 10 of the 19 non-completing cases in Guangzhou had Crohn’s disease (often complicated by intestinal stenosis or ulcers), which is a known risk factor for prolonged CE transit (Liao et al., 2024; Yang et al., 2022; He et al., 2017; Wang et al., 2020). The potential reasons of the above difference might be the composition and degree of the disease. For example, the seven patients who did not complete CE examination in this study included 1 Crohn‘s disease, 2 Meckel’s diverticulum, 1 Henoch-Schonlein purpura, and three non-specific inflammation. However, the 19 children without completion of CE in Guangzhou included 10 Crohn‘s disease, six non-specific ulcer, 1 Peutz-Jeghers syndrome and two negative results.

Average SBTT of children who swallowed the capsule and underwent endoscopic placement of capsule was 4h11min and 4h16min respectively, with only 5min time difference. Such difference was nearly 3 h (4.8h and 7.68 h) according to the previous study from Xi’an (Yang et al., 2022). Notably, CE’s small bowel transit time (SBTT) varies significantly across regions—for example, the 5-minute difference between oral and endoscopic placement groups in our study contrasts sharply with the nearly 3-h difference (4.8 h vs. 7.68 h) reported in Xi’an (Yang et al., 2022). This regional variation may be attributed to multiple factors: (1) differences in dietary habits (e.g., fiber intake) and physical activity levels among children in different regions; (2) variations in disease spectrum (e.g., higher prevalence of intestinal inflammation in some regions may delay transit); (3) age distribution (SBTT is known to increase with age in children); and (4) differences in bowel preparation protocols (e.g., dosage of laxatives or timing of administration). Other studies indicated that SBTT was prolonged with age and the degree of small bowel stenosis in adults (Tominaga et al., 2020), but was significantly shortened in obese patients (Omori et al., 2022), suggesting that age and pathophysiological conditions had an effect on SBTT of CE. It was further found that the transit time was statistically longer in the small bowel ulcer group than in the normal and small bowel inflammation groups, but the conclusion was controversial (Yang et al., 2022), so larger sample size and stratified analysis are required.

The variation in disease detection rates (76.82% in our study vs. 31.81% in Xi’an, 55.6% in Shanghai, 90% in Zhejiang) is speculated to stem from two key factors: (1) differences in disease spectrum composition—for example, Zhejiang’s study included a higher proportion of children with suspected Crohn’s disease (a condition with distinct endoscopic features), while our study had more cases of non-specific inflammation (milder, less overt lesions); (2) differences in the stringency of CE indications—our study included only children with high clinical suspicion of small bowel disease (e.g., refractory abdominal pain, unexplained anemia), whereas other studies may have had broader inclusion criteria, leading to more negative results (Liao et al., 2024; Yang et al., 2022; Ma et al., 2009; He et al., 2017). In this study, the frequency of detected diseases in descending order was non-specific inflammation, Crohn‘s disease, Meckel’s diverticulum, Henoch-Schonlein purpura, intestinal parasitic diseases, collagenous gastritis, Peutz-Jeghers syndrome, lymphangiectasia, and BRBNS. Among them, intestinal non-specific inflammation and Crohn‘s disease were the most common positive findings, similar to Guangzhou and Xi’an, while Crohn‘s disease was the primary finding in Zhejiang (Yang et al., 2022; Ma et al., 2009; He et al., 2017), and gastrointestinal bleeding and Crohn‘s disease were the most common in adults (Kharazmi et al., 2020).

In this paper, we analyzed the detected diseases in children of different age groups. In addition to non-specific inflammation as the primary finding in all age groups, negative results were the most common in 0–6 years group; In the 7–12 years age group, after non-specific inflammation (the most common finding), normal small bowel mucosa (28 cases) and Crohn’s disease (16 cases) were the second and third most frequent results. In the 13–18 years group, Crohn’s disease (13 cases) ranked second, followed by normal mucosa (8 cases). This indicates that school-aged children and adolescents are at higher risk of Crohn’s disease compared with preschool children. It could be seen that compared with preschool children, school-aged children and adolescents were most vulnerable of Crohn‘s disease (Légeret et al., 2022; Kaul et al., 2024; Oka et al., 2023). None had ulcerative colitis” was deleted, as SBCE is not indicated for ulcerative colitis diagnosis and this information is irrelevant to the study’s focus (Oka et al., 2023). Previous study indicated that 30% of Crohn‘s disease would involve the small intestine. All 29 children with Crohn’s disease had positive findings in our study, with different degrees of small intestinal mucosal erosion, ulcer or proliferative lesions. The diagnosis needed to be combined with medical history, laboratory, colonoscopy and pathological results for comprehensive analysis. The 4 cases of Henoch-Schonlein purpura were all boys, mainly 7–12 years. CE found that multiple erosions and ulcers were present in the jejunum and ileum in 3 cases. According to prior research, the detection rate of Meckel’s diverticulum was 41% in children who underwent CE for hematochezia (Wu et al., 2020). While in our study, such rates were 13.04% and 15.79% in children with hematochezia and anemia, respectively, suggesting the important diagnostic value of CE for Meckel’s diverticulum. Besides, four intestinal parasitic diseases were identified in 0–6 and 7–13 years groups, with abdominal pain as the main symptom, so parasitic infection was still worthy of attention among preschool and school-aged children., combined with intractable anemia, characteristic lip black spots, cutaneous hemangioma, unexplained long-term edema, hypoproteinemia and other medical history, disease diagnosis is always not difficult. Therefore, small intestinal diseases in children have distinct age and gender characteristics, as well as disease stratification (Ou et al., 2023), CE can be targeted for screening.

The most common complication of CE examination remains capsule retention (Rosa et al., 2023), with a retention rate of 0.36% in childrenand 2% in adults. No capsule retention was observed in our 151 pediatric patients, despite 29 cases of Crohn’s disease—a condition associated with a risk of capsule retention due to intestinal stenosis. This may be attributed to strict patient selection: children with known or suspected intestinal stenosis (a key risk factor for retention) were excluded from the study per the inclusion/exclusion criteria.

There have some limitations in this study. First, there existed possible false negative results because CE was unidirectional in the bowel and imaging at one time. Second, although two gastroenterologists independently analyzed the images, potential selection bias may exist in the extraction and classification of diagnostic findings—for example, inter-observer variability in defining “non-specific inflammation” or distinguishing early Crohn’s disease from other inflammatory lesions. Last, the design of single-center retrospective study might limit our sample size. Thus, a multicenter, large-sample prospective study is needed to further validate our conclusions.

In summary, unlike MR enteroclysis (MRE) requiring children’s sedation and immobilization and CT enteroclysis (CTE) with radioactivity and high equipment costs, CE has unique advantages such as intuitiveness, easy operation, safety, and non-invasiveness in the diagnosis of small intestinal diseases. Therefore, CE has good practical application value and can be further promoted in pediatric clinical practice. In the future, CE is expected to achieve biopsy and treatment function, further improve the diagnosis and treatment efficacy, better serve children, and create good social and economic benefits as well.

## Data Availability

The original contributions presented in the study are included in the article/supplementary material, further inquiries can be directed to the corresponding authors.
